# Development and construction of a portable wind tunnel for investigating wind erosion through the application of photogrammetry techniques

**DOI:** 10.1038/s41598-024-84496-9

**Published:** 2025-01-02

**Authors:** Noredin Rostami, Maryam Rabbani, Saman Esmaeilbeigi, Kamyar Hassanpour, Mohammad Hossein Shahmoradi, Mohammad Najafishoa, Zahra Ghobadian, Masood Varshosaz, Mehdi Norianfar

**Affiliations:** 1 Department of Range and Watershed Management, Faculty of Agriculture, Ilam University, Ilam, Iran; 2 Department of Electrical Engineering, Ilam University, Ilam, Iran; 3 Geomatics Engineering Faculty, K.N. Toosi University of Technology, Tehran, Iran; 4 Independent Scholar, Tehran, Iran; 5 Department of English Language and Literature, University of Tehran, Tehran, Iran; 6 Artificial Intelligence Institute, University of Shaoxing, Shaoxing, China; 7 Department of Electrical Engineering, Faculty of Engineering, Razi University, Kermanshah, Iran

**Keywords:** Wind tunnel, Portability, Photogrammetry, Motor speed control, Environmental impact, Natural hazards, Energy science and technology, Engineering

## Abstract

Climate change is one of the most crucial issues in human society such that if it is not given sufficient attention, it can become a great threat to both humans and the Earth. Due to global warming, soil erosion is increasing in different regions. Therefore, this issue will require further investigation and the use of new tools. In this paper, a portable wind tunnel was designed and built for wind erosion studies using photogrammetry, which is a novel technique. This instrument consists of a working section, a fan, a portable voltage source inverter to control its angular speed, and a honeycomb to straighten the air flow in the working section. The eroded volume of soil is measured using photogrammetry by producing two 3D models and point clouds before and after the soil erosion test and calculating their volume changes. The results show that a 0.175 mm check distance precision is achievable under convergent imaging and with sufficient number of accurate control points which this value indicates the discrepancy between the anticipated and measured lengths of all the check distances. In addition, the features of this portable wind tunnel guarantee its easy portability, and its transparency enables the measurement of the threshold friction velocity. Additionally, this instrument, as an invention, has been registered at the Intellectual Property Center of Iran.

## Introduction

With the increasing impacts of climate change and recurring droughts^1,2^, arid and desert regions expand each year, turning wind erosion and dust storms into widespread issues. On the other hand, the presence of scattered and sometimes contradictory data can lead to confusion in decision-making and subsequent implementation of various projects for the rehabilitation of arid and desert areas. Therefore, it is necessary to use appropriate tools for conducting studies on soil wind erosion and to obtain valid and reliable data. The device commonly used for such studies is known as a wind tunnel. In this device, wind is blown at an adjustable speed onto a sample of soil, and various methods are used to measure soil erosion. The capabilities of a wind tunnel include estimating soil loss, determining the threshold of wind erosion, and examining the effects of different soil covers, vegetation, and more on soil erosion. Wind tunnels with various capabilities have gained significant attention from researchers in recent decades. In addition, most research papers preferred using laser scanners to scan soils due to their high accuracy. However, the price of laser scanners is exorbitant. In this paper, we introduce a cost-effective and highly accurate method for 3D scanning using Photogrammetry. Photogrammetry is a technique used to obtain reliable measurements and 3D models from photos. By taking multiple overlapping images of an object or area from different angles, photogrammetry software analyzes the photos to create a detailed 3D representation. It’s commonly used in fields like surveying, mapping, architecture, and environmental monitoring. The process involves capturing images, processing them with specialized software, and extracting geometric information to produce accurate models.

In Iran, for the first time in 1991, a device for measuring wind erosion was designed and built known as the Wind Erosion Meter (W.E.Meter). This device is composed of three parts: a wind generator capable of producing wind speeds of approximately 12 m/s at a height of 25 cm above the ground, a metal channel for contact with the soil, and a plastic collector for collecting sediment^3^.

In another study, Hong et al*.* 2014^4^ proposed a model for predicting wind erosion through measurements taken using a portable wind tunnel. This model is a modified form of the wind erosion equation (WEQ) and reflects short-term erosion using quickly measurable and simple factors. A portable wind tunnel in another research^5^ consisting of three parts: a 2-horsepower jet fan generator capable of producing wind speeds from 0 to 22 m/s at a height of 25 cm above the ground, a working section with dimensions of 0.3*0.3 m, a test area with dimensions of 1*0.3 m, which was placed directly on the surface of the undisturbed soil, and an 8-m plastic sediment trap. In another study in the Columbia Plateau region of the Pacific Northwest United States to assess the impact of management practices on erosion, a portable wind tunnel 7.3 m in length, 1.2 m in height and 1 m in width used to validate the RWEQ and SWEEP models in simulating soil loss and PM10 emissions^6^. Also for evaluation of changing land use management in Ebro Basin a field experiments with a portable wind tunnel^7^, average wind speed of 8 m/s 15 cm above the ground and a 3 m rectangular working section with dimensions of 0.7*0.7 m, conducted,.

Additionally, field experiments using a portable wind tunnel^8^ conducted on wind erosion with a rectangular cross-sectional area measuring 0.5*0.4 m and a working section extending up to 5 m. The wind speed can vary from 0 to 22 m/s. At the end of the tunnel, a sediment collection trap with a width of 3 m, length of 5 m, and depth of 1 m is used.

**Fig. 1:**
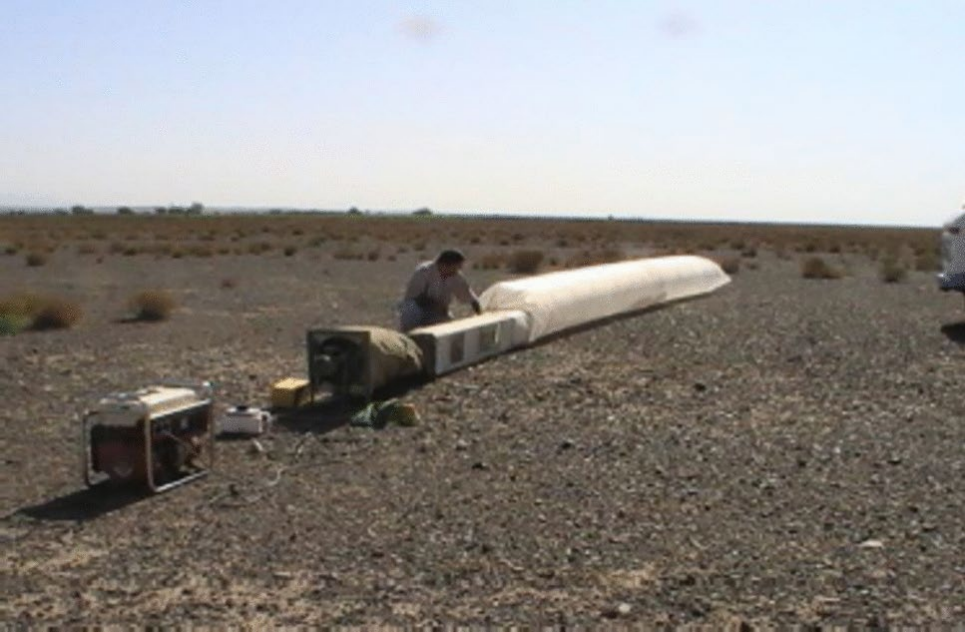
The wind tunnel constructed by Ayazi et al*.,* (2016)^9^.

A portable wind tunnel^9^ used to investigate the sediment-producing potential of geomorphological landforms in Kashan, Iran. The device included a portable power generator, a wind speed control switch, an air transfer interface from the fan to the tunnel, the experimental tunnel body, and a sediment collection chamber. The tunnel had a transparent glass window for observing all internal interactions (Fig. 1). These studies showcase the versatility and significance of portable wind tunnels in advancing our understanding of wind erosion under diverse environmental conditions.

Table 1 present some relevant studies about wind tunnel and show that how this instrument can be applied in various fields.

**Table 1:** Examples of field studies using wind erosion tunnel.

Study field	Reference
Evaluating the polyvinyl acetate effect on wind erosion of different soils	Movahedan et al*.,* (2011)^10^
Sediment-producing potential in geomorphological landforms using a wind tunnel and IRIFR1 model in Gonabad County, Iran	Mohammadnia et al*.,* (2019)^11^
The impact of wind speed and particle size distribution on sediment transport due to wind erosion	Mahmoudabadi et al*.,* (2012)^12^
The influence of soil properties on wind erosion intensity in various regions of Kerman Province, Iran	Shahabinejad et al*.,* (2020)^13^
Wind erosion susceptibility of soil against various salts using a wind erosion measurement device	Ekhtesasi et al*.,* (2003)^14^
Determining the best combination of steel slag as mulch for preventing wind erosion in the eastern region of Isfahan, Iran	Karim-zadeh et al*.,* (2012)^15^
The role of biological windbreaks in creating microclimates in desert areas of Dehloran, Iran	Mirhasani et al*.,* (2021)^16^
The IRIFR1 wind erosion prediction model and its comparison with direct measurements using the W.E.Meter device in the Isfahan Segzi Plain, Iran	Ekhtesasi et al*.,* (2016)^17^
The impact of different plant densities on wind erosion in a wind tunnel	Miri et al*.,* (2016)^18^
Evaluation of an empirical model in a wind tunnel using image processing	Fattahi et al., (2014)^19^
Measurement and prediction of soil erosion in drylands using a portable wind erosion tunnel	Hong et al., (2014)^4^
Evaluation of RWEQ and SWEEP models in simulating soil loss and PM10 emissions from a portable wind tunnel	Huawei et al., (2017)^20^
Designing the aerodynamics of a portable wind tunnel for soil erosion and dust research	Pietersma et al*.*, (1996)^21^
Wind erosion in the Central Abruzzo Basin under land-use change, field experiments with a portable wind tunnel	Fister et al*.,* (2009)^7^
Evaluation of a small portable wind tunnel for energy harvesting	Tian et al., (2021)^22^
A small portable wind tunnel for educational purposes	Ismail et al*.*, (2022)^23^
Assessment of dust emission potential using a small wind tunnel in Bakhtegan Playa, Iran	Karimzadeh et al*.,* (2019)^24^
Bayesian belief network model for wind erosion hazard assessment: an experiment with wind tunnel data	Kouchami-Sardoo et al*.,* (2019)^25^
Wind tunnel and field assessment of different dust suppressants	Preston et al*.,* (2020)^26^
Image analysis for soil erodibility assessment in a wind tunnel	Asensio et al*.,* (2018)^27^
Dynamic processes of dust emission: A study based on a portable wind tunnel in China	Zhang et al*.,* (2022)^28^
Wind erosion of arenosols (sandy soils) and driving factors during dune stabilization: A wind tunnel experiment	Chen et al*.,* (2022)^29^

The goal of this project was to construct a highly accurate portable device for measuring soil erosion. This method aims to meet the needs of laboratory equipment and research centers related to wind erosion and soil erodibility measurements and also biological and chemical mulchs and treatment for soil conservation. This device has been registered with a unique ID of 140,250,340,003,000,302 and a verification code of 964,168 in the Intellectual Property Center of Iran.

## Materials and methods

**Fig. 2:**
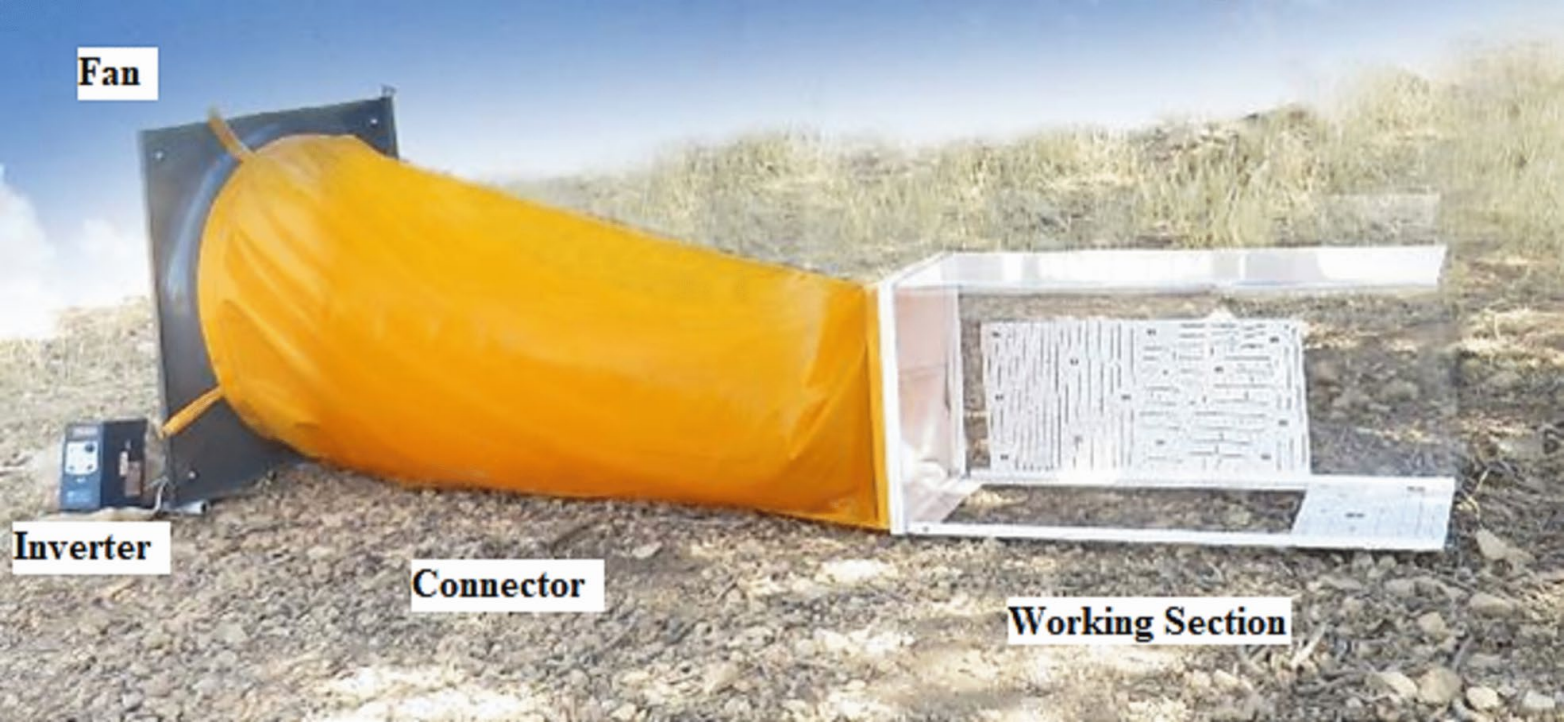
An illustration of the designed and constructed wind tunnel. This wind tunnel device consists of a portable fan and generator, a voltage source inverter for voltage and wind speed regulation, a coaxial connector for transferring airflow from the fan to the tunnel, a mirrorless camera for taking suitable images in both epochs and a transparent Plexiglas body.

This wind tunnel device, which was designed and invented for wind erosion studies, consists of various components, including portable fan and generator, inverter, coaxial connector, mirrorless camera (Table 2) and besides having a high-resolution camera for this project, a large pixel size is also necessary and transparent Plexiglas body (Fig. 2). In what follows, each of these components and the techniques employed will be described in more detail.

**Table 2:** Attributes of the used camera.

Camera model	Resolution	Focal length	Pixel size	Type
EOS M50 Mark II	6000 × 4000 pix	22 mm (fix lens)	3.7 μm	Mirrorless

### Body design

**Fig. 3:**
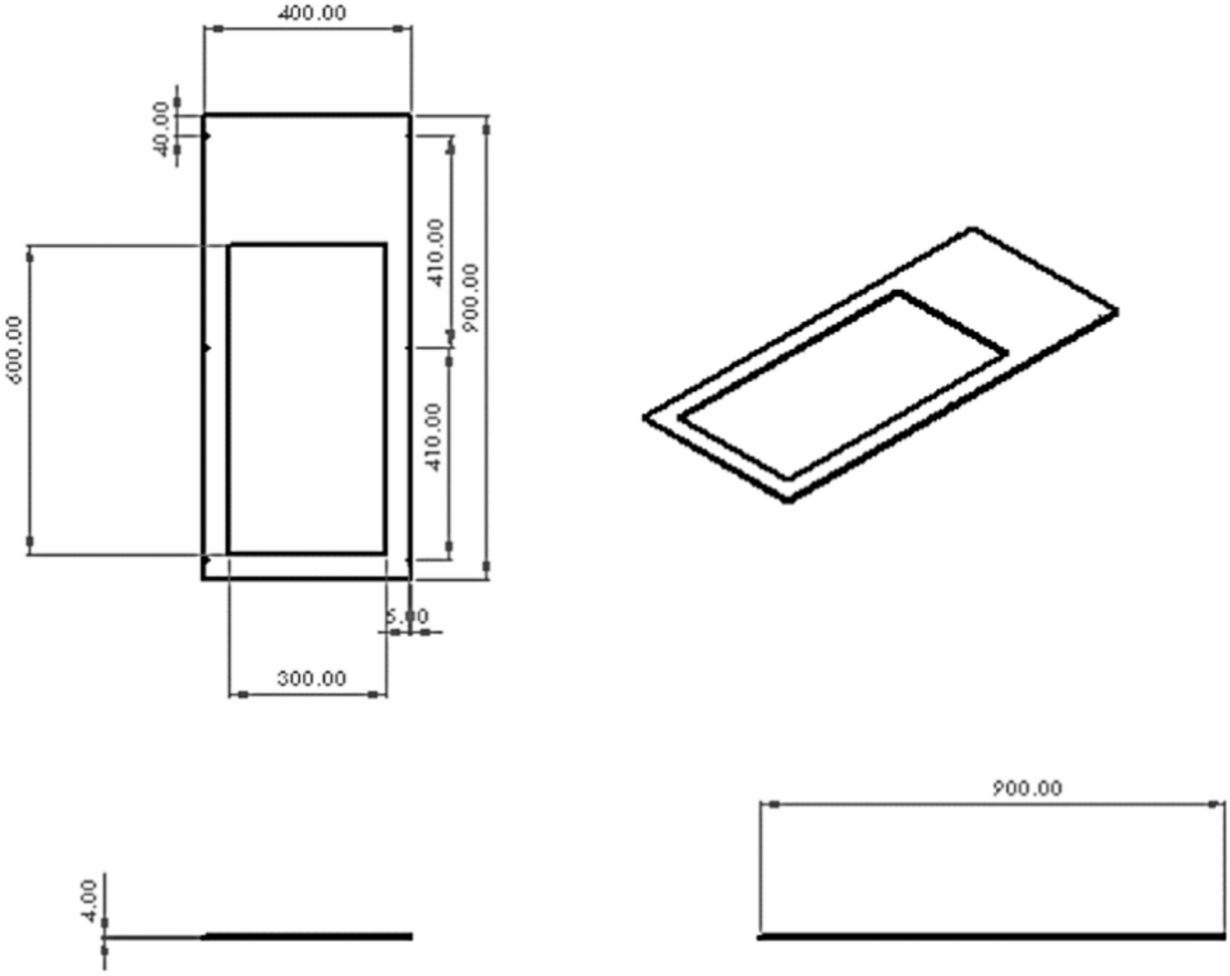
A view of the designed cutout sheet in the working section using AutoCAD software. A steel sheet measuring 40*90 cm with a thickness of 4 mm was used as the surface that sits on the soil. From this sheet, a rectangular piece measuring 30*60 cm, with 5 cm of space from three sides and 25 cm from the fourth side, was cut. This cutout will serve as the “side sheet” of the device. By placing the bottom sheet on the soil, the empty rectangular part in the middle of it will form the soil sample under test, which will be exposed to controlled wind flow at a specified speed. Two 90 cm aluminum rails are placed on both sides of the bottom sheet in a longitudinal fashion.

To achieve the project’s main goals, which include high precision in calculating the volume of eroded soil, the portability of the device and transparency of the working section, a wind tunnel was constructed (Fig. 3). For the top part of the tunnel, a transparent Plexiglas sheet with a thickness of 5 mm and dimensions of 122*91 cm bent at a 90-degree angle from the midpoints of two longitudinal lines with a distance of 40 cm from the sides is used.

**Fig. 4:**
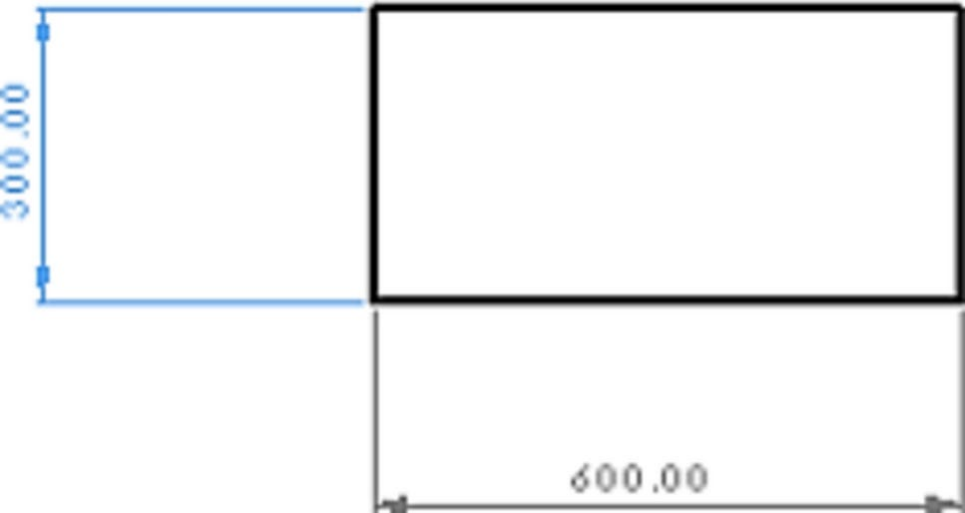
An illustration of the side sheet design in the working section for the installation of targets which help to precise execution of the photogrammetric process.

For precise execution of the photogrammetric process, it is necessary to install several targets along both the horizontal and vertical axes to independently position and strengthen the network around the sample area. To install these targets, a vertical sheet is welded to the side of the bottom sheet. The distance of this sheet from one side of the bottom sheet was 5 cm, and from the other side, it was 25 cm (Fig. 4).

**Fig. 5:**
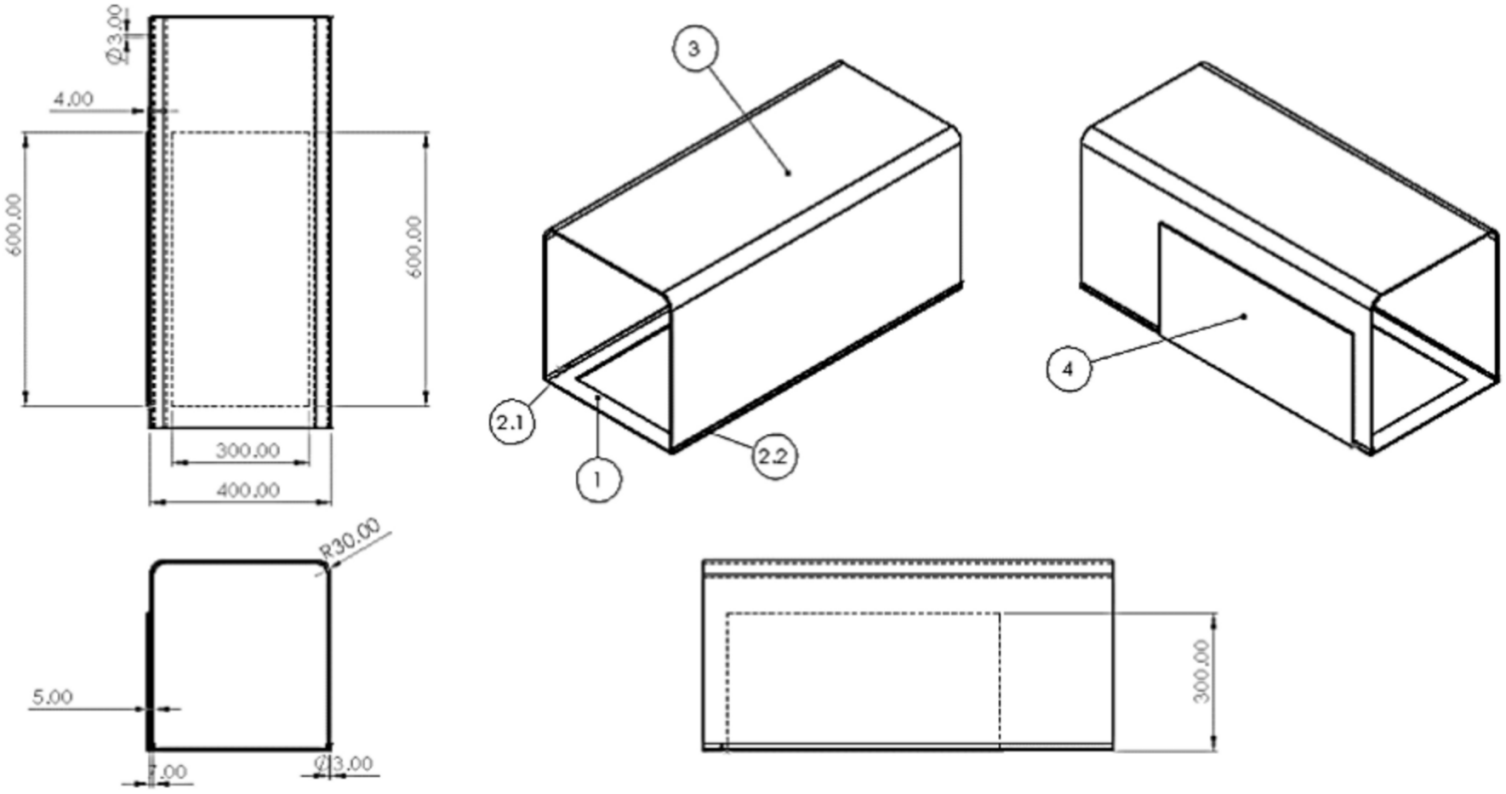
A view of the working section designed in AutoCAD software. Testing the soil sample is the main goal of wind tunnel and this transparent working section show the process of wind erosion and also its threshold as the minimum friction velocity required to initiate movement of soil particles.

The sides of the bent Plexiglas sheet fit inside the two aluminum rails, creating the “working section” of the wind tunnel (Fig. 5).

**Fig. 6:**
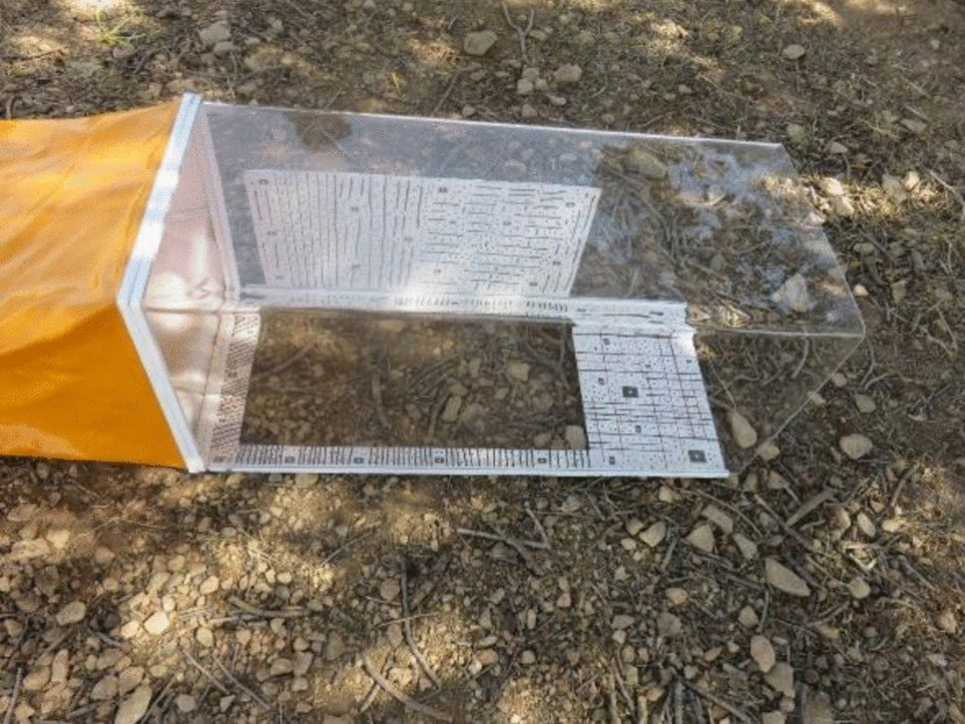
The body and working section of the wind tunnel which during this part the soil sample and also targes are visible and user can take images before and after wind blowing test.

The connection between the fan and the working section is established by a 1.5-m-long canvas duct. Inside this channel formed by the canvas duct, a 4-cm-thick cardboard honeycomb network is installed within a square aluminum frame to ensure a uniform airflow speed. Since an axial fan is used in this design, the air velocity at the fan’s center is at a minimum, and that at the edges of the frame is at a maximum. The honeycomb network helps to equalize the airflow speed throughout the canvas duct and, consequently, across the entire surface of the working section. (Fig. 6).

### Electrical section

**Fig. 7:**
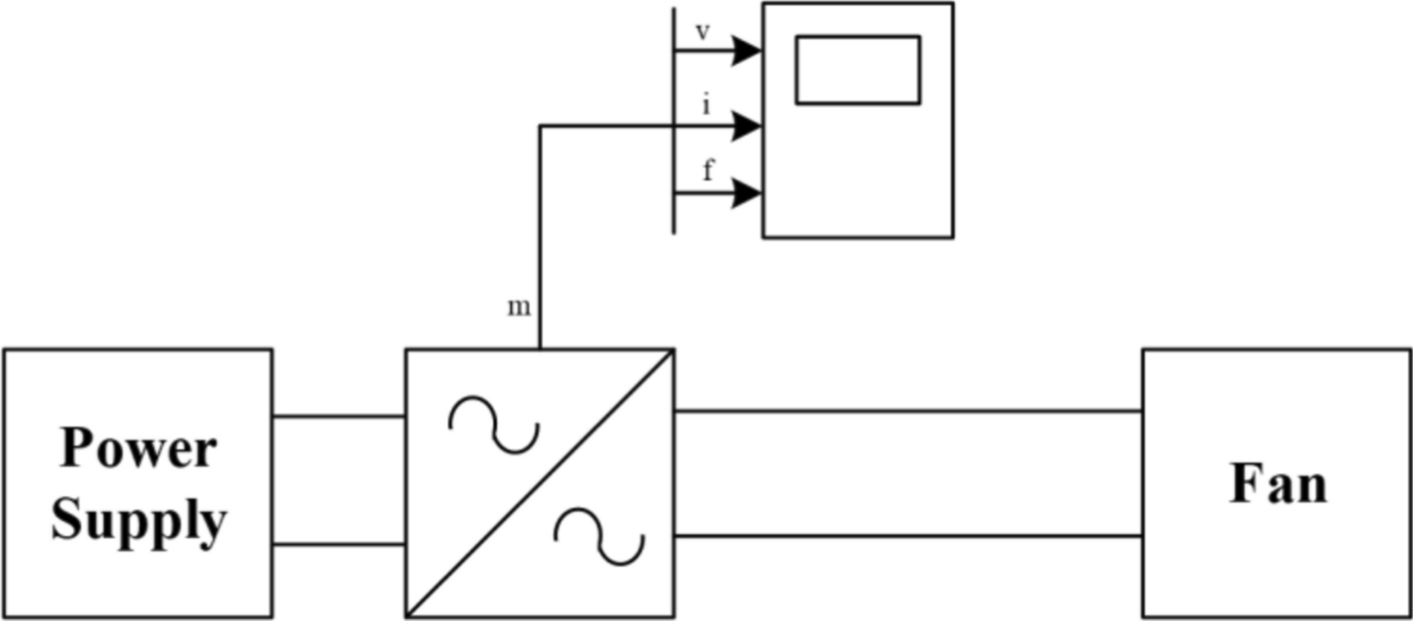
Schematic of the main electrical equipment.

One of the important sections in the proposed scheme is the electrical section. In this section, the required wind speed (input air of the wind tunnel) to check the soil erosion is provided by the electric components. The schematic of the electrical section is shown in Fig. 7. Here is a 220V power supply with a frequency of 50Hz, which supplies the fan by a single-phase inverter. The basic input and output parameters of the inverter such as voltage (v), current (i), and frequency (f) are displayed by measurements so that the user knows the process. Some of the most important components of this section are given below.

- Single-phase electric motor with a power of 3 kW.
- Single-phase blower fan with an air output of 12,000 m^3^/h and a maximum speed of 17 m/s. This fan utilizes a capacitor-run induction motor, and the presence of a capacitor increases the starting torque, which is crucial for single-phase motors with low starting torque.
- A single-phase inverter for controlling the fan’s speed using the voltage and frequency control (V/F) method. The inverter is used to prevent damage to the motor and increase its lifespan.

**Fig. 8:**
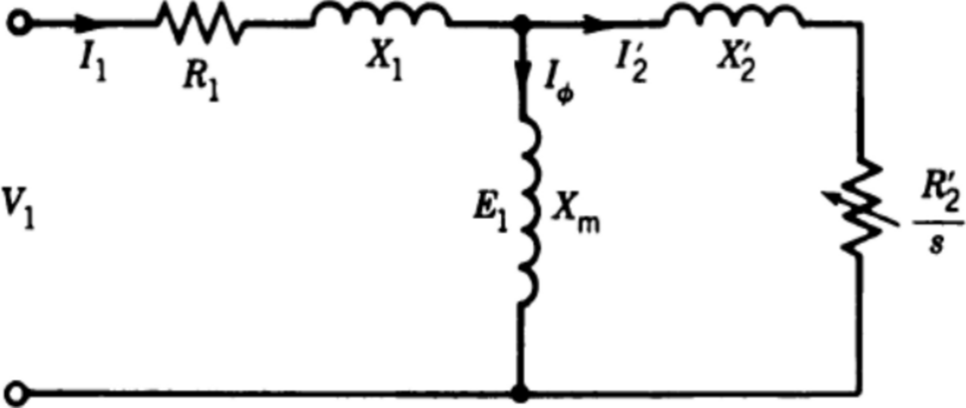
The circuit schematic of an induction motor.

Because the proposed work requires different output air volumes (wind speed) to check the soil erosion, it should be possible to change the volume of air output from the fan in the electrical section so that no damage is caused to it. There are different methods for this, including changing the voltage and the frequency of the source, separately, but these methods alone cause the fan to heat up and damage it. The circuit schematic of an induction motor used here as a fan is shown in Fig. 8.

The induced voltage equation is presented in the Eq. 1. In this equation, E, f, N, and φ represent voltage, frequency, number of winding turns, and maximum flux, respectively.

1 $$E_{1,rms} = 4.44fN\varphi_{m}$$

According to Eq. 1, it can be concluded that many parameters such as flux and frequency can affect the voltage. In the used fan, the specifications of which are given in Table 3, if we only increase the voltage, the speed of the fan and therefore the volume of the output air (motor speed) will increase, but this will increase the flux, saturate the fan core (core saturation), and eventually heat the system. Also, frequency change alone is less used because of the problems it brings. Therefore, in this work, a single-phase inverter is used to control the simultaneous voltage and frequency. So that as the voltage increases, the frequency also changes and remains constant according to the flux formula (v/f), and the fan is not damaged. The specifications of the single-phase inverter used are presented in Table 4.

**Table 3:** The specifications of the fan used in the studied wind tunnel.

Parameter	Value
Model	VIE-60R4S
Company	Damandeh
Blades radious	30 cm
Voltage	220 v
Frequency	50 Hz
Rated current	3.6 A
Output power	780 w
Speed	1380 round per minute (rpm)
Air valume	12,000 m^3^/h

**Table 4:** The specifications of the single-phase inverter used in the studied wind tunnel.

Parameter	Value
Model	PENTAX-DSI-200
Company	PENTAX
Dimensions	127*170*84 mm
Voltage	220 v
Frequency	0–50 Hz
Input rated current	14.1 A
Output rated current	7 A
Rated power	1500 w

### Photogrammetry section

**Fig. 9:**
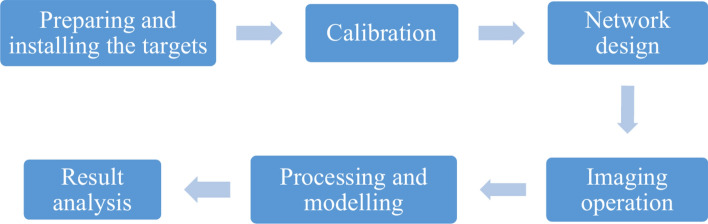
Stages of completing a close-range photogrammetry project.

Photogrammetry can be defined as the science, art, and technology of acquiring reliable information about objects and phenomena on the Earth’s surface through the processing of data obtained from it by a camera without direct contact with the object^30^. Photogrammetry methods are often classified into three categories: terrestrial photogrammetry, aerial photogrammetry, and close-range photogrammetry. Close-range photogrammetry is a branch of photogrammetry that involves the measurement, interpretation, and 3D modeling of objects and scenes based on images obtained from a short distance^31^. Recent advancements in digital camera technology, although not primarily designed for photogrammetric purposes, have significantly contributed to the field of photogrammetry, particularly in recent years. In recent projects, researchers tend to prefer using LiDAR laser scanners for quick and accurate scanning due to their high speed. However, LiDAR devices are quite expensive, with prices starting at $2,000. In contrast, our proposed method, which utilizes photogrammetry techniques and a stable digital camera, achieves the same results without the need for costly equipment.In this project, one of the objectives is to model and analyze changes in the ground surface over an area of 1800 cm^2^ at different time intervals and under specific wind speeds. These variations are measured using the close-range photogrammetry technique. In other words, by placing this wind tunnel in the study area to measure soil loss with different compositions at different time intervals corresponding to varying wind speeds, data are collected and compared to previous conditions (Fig. 9). In this project, the implementation of an off-line photogrammetry system was addressed. Off-line systems refer to industrial monitoring and modeling of objects where the measurement and data collection phase is separate from the image measurement phase.

However, we need to evaluate our proposed method using a more accurate technique than photogrammetry. Due to limitations in accessing a highly accurate LiDAR laser scanner, we used control (for scaling) and check (for evaluation) scale bars, which were measured with high precision (5 µm) using a digital caliper.

Subsequently, the steps and important considerations in each of the stages in Fig. 8, as well as the overall conditions and factors affecting the accuracy of the results, will be discussed.

### Preparation and marking

**Fig. 10:**
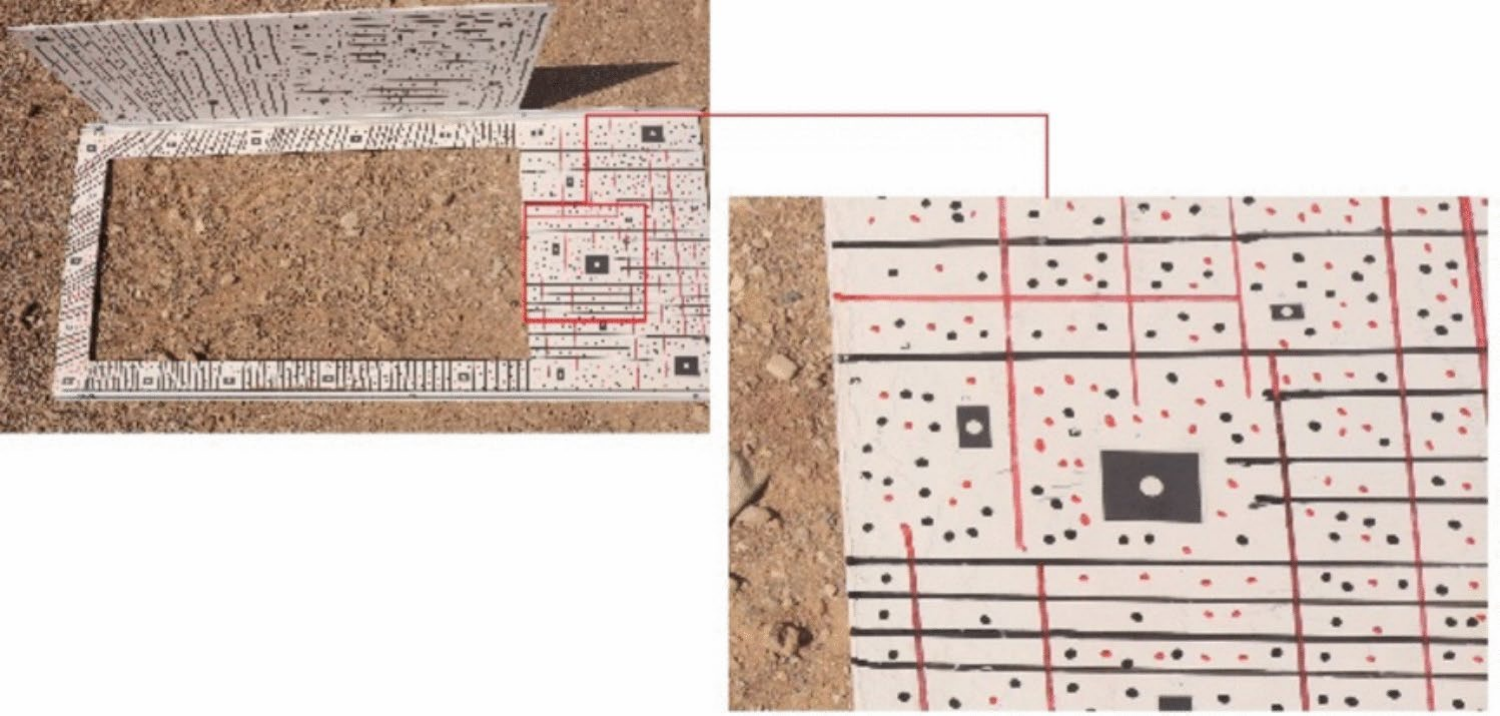
Targets installed on the working section body.

To ensure proper photographic conditions, it is necessary to create suitable environmental conditions. For example, the object, the imaging environment, and the conditions affecting accuracy should be optimized to achieve the best results. To establish a unified metric coordinate system and perform the calibration process, control points in the object space must be created. An example of these control points, known as the black and white rectangular targets used in this study, is shown in Fig. 10.

As shown in Fig. 9, the manual targets facilitate the identification of the target center using smart image processing algorithms due to the sharp contrast between the black and white background and the center. The target marking step is carried out before the imaging operation. Depending on the chosen method for capturing the object and the required precision, target marking is performed while considering the following:

- The number of targets used in the network depends on factors such as the minimum required distance for optimal modeling and bundle adjustment calculations, cost considerations, maximum target density, software limitations, and object complexity^31^.
- The accuracy of measurements and the estimation of calibration parameters depend greatly on the number and distribution of targets in the three-dimensional object space and the two-dimensional image space. Empirically, a minimum of 25 targets is recommended, with an average of approximately 20 targets per image^30^ in close-range photogrammetry projects, the dimensions of the object or working area are approximately 2 ${m}^{3}$
- The suitability of a target depends on its material, durability, reflectivity, dimensions, shape, symmetry, and contrast.

**Fig. 11:**
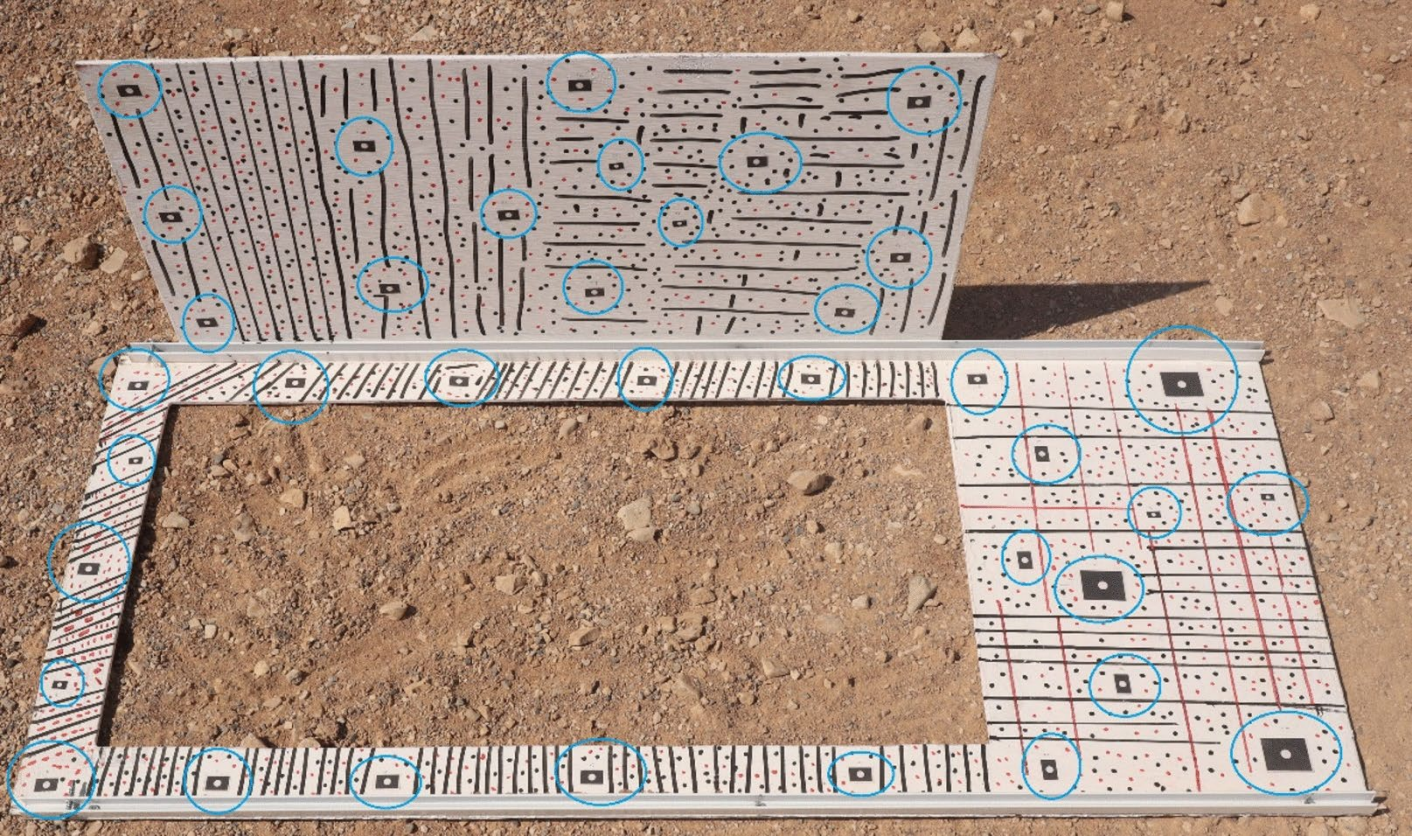
The targets used to create the link for control and check lengths.

Therefore, considering this condition, 37 control points (targets) were designed and distributed on the wind tunnel body. Using a digital industrial caliper tool, the distances between each of the targets (measured three times for scale) were measured for scaling purposes (Fig. 11).

### Calibration

Calibration refers to defining the path of light inside the camera based on camera parameters, which needs to be done for the following reasons: A) The light path inside the camera is not known (requiring the determination of internal camera parameters such as principal distance and principal point coordinates). B) Often, the lenses used are not standard, so it must be determined how light passes through these lenses exits (distortion factors). If calibration parameters are calculated with high precision and are included in the transformation equations, such as the collinearity condition^28^, a good relationship between two spaces can be established, and the final coordinates can be estimated with high accuracy.

In this project, multiple tests were performed to model and determine calibration parameters, which are beyond the scope of this article’s objective. To conduct soil modeling tests using photogrammetry, a test field was initially constructed, from which 45 images were taken (considering network design guidelines). In the next step, the image coordinates of the target points were measured with an accuracy of 0.05 pixels, and bundle adjustment computations^32^ were performed. To transfer the coordinate system of the generated model to a local metric coordinate system, six scale bars, measured with a digital industrial caliper tool with an accuracy of 5 microns, were used (Fig. 11). Australis software version 7^33^ was used for measurements and photogrammetry computations in this research. Finally, the internal and external camera parameters, as well as the calibration parameters, along with the three-dimensional positions of the target points (hereafter considered control points), were obtained. Next, to check the internal strength of the camera, the calibration parameters and the changes in the internal elements of the camera were recalculated. In other words, in the working section in the laboratory environment, the parameters of the camera calibration and the position of the centers of the targets were determined by converging imaging and creating a strong network. As mentioned, the purpose of recalculating the calibration parameters is to check the geometric strength of the camera, with the difference being that the images were taken from the targets of the horizontal and vertical planes of the wind tunnel body. The process of recalibration calculations was performed by taking 45 converging images of the body of the wind tunnel working section in the Australis software. This test showed that the Canon M50mark2 (Table 2) camera has good body strength because the amount of change in the internal elements of the camera was almost constant in both tests. Consequently, the camera calibration parameters were obtained from Australis as initial values for self-calibration in Metashape (Agisoft Metashape is a software used for photogrammetry workflows).

### Network design

Network design involves decisions regarding camera specifications, the number of camera stations, the number of image repetitions, geometric network strength, image observation accuracy, and related topics. The goal of designing close-range photogrammetry networks is to achieve criteria such as accuracy, reliability, and cost-effectiveness^34^. When measuring large or complex objects, meeting these criteria becomes more challenging. Therefore, the design of close-range photogrammetry networks is of particular importance in such cases. The general relationship for error propagation (propagation of uncertainty) can serve as a basis for designing close-range photogrammetry networks^35^ (Eq. 2) :

2 $${\sigma }_{c}=\frac{q.s.\sigma }{\sqrt{k}}=\frac{q.d.{\sigma }_{a}}{\sqrt{k}}$$

In this equation, ${\sigma }_{c}$ represents the average standard deviation of the XYZ coordinates of the object points, q is the design factor or geometric strength coefficient of the network, k is the number of average images taken at each camera station, s is the scale factor, which is equal to the ratio of d (average distance from the camera to the object) to c (principal distance of the camera), σ (10% pixel size) is the average error in the xy coordinates of the image points and ${\sigma }_{a}$ is the corresponding angular error. Hence, measurement accuracy depends on three parameters: camera angular resolution (the ratio of the standard measurement error in image coordinates to the camera’s focal length), network geometric strength, and image scale. In theory^36^, measurements can be made with only two images, but it is recommended to use at least 4 to 6 images for each object surface, considering the field of view of the camera^37^.

To achieve adequate accuracy, it is empirically recommended that a minimum of 12 points be seen in each image. However, it should be noted that in networks with high geometric strength, the measurement accuracy of the points is independent of the density of the targets, and it has been found through experience that measuring more than 40 points in each image will not significantly improve accuracy^38^. It is worth mentioning that in addition to network design, the dimensions of the images and the resolution of the imaging system have a significant impact on the accuracy of the photogrammetric system. For industrial photogrammetry applications with required accuracies ranging from 1:20,000 to 1:200,000, digital cameras with high resolution in the range of 2 to 10 microns are required^39^.

### Image acquisition

**Fig. 12:**
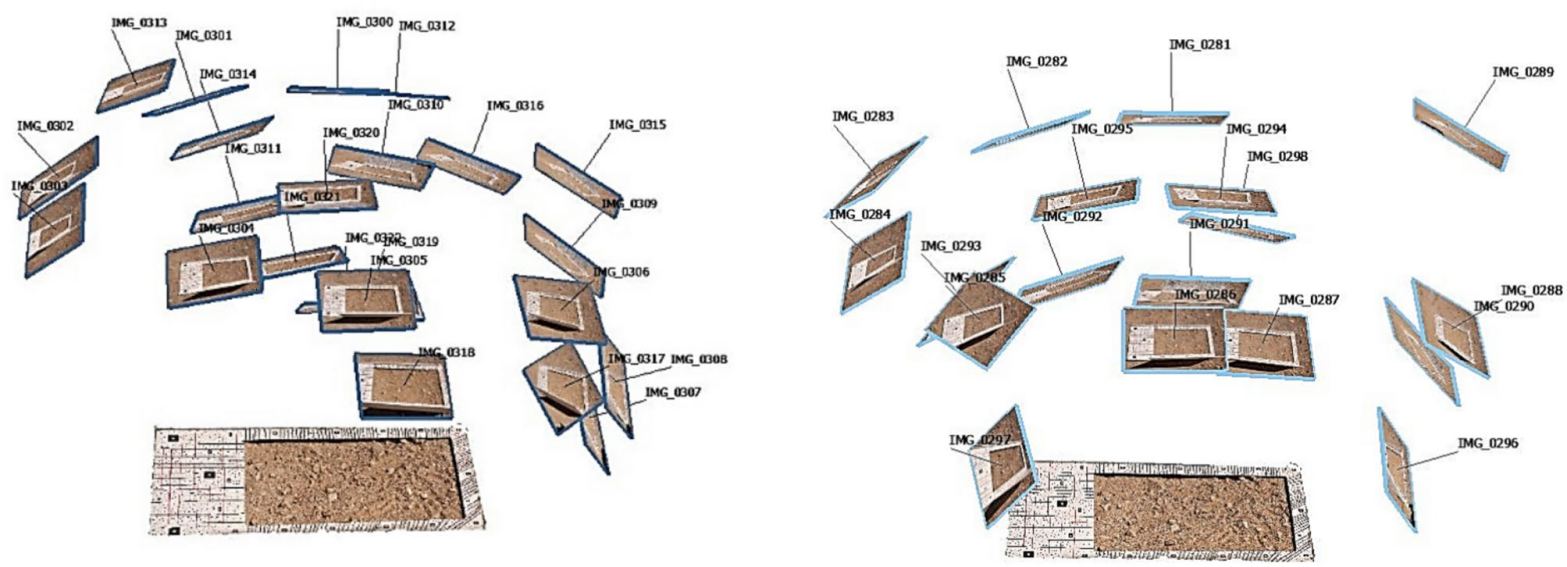
The position of images captured for the first (left) and second (right) epochs.

Image acquisition is performed in a convergent manner to ensure that all sections of the soil are captured or scanned so that a high-quality 3D model can be reconstructed (Fig. 12). Therefore, with the installation of the wind tunnel body in the field, image acquisition of the soil was performed in two phases: before and after the soil surface changes (due to the simulated wind erosion). In other words, to create a 3D model of the soil, 18 and 23 images were captured in the first and second epochs, respectively. It is essential to comply with spherical geometry and ensure convergence in data acquisition for in this type of project and the number of images in each epoch may differ.

### Processing and modeling

By performing bundle adjustment on the images from both epochs simultaneously, the positions and orientations of the imaging stations were determined. The calibration parameters calculated in the camera calibration step were used (camera calibration parameters obtained through australis) as initial values for the calibration parameters in this project to increase accuracy and convergence. Also, a self-calibration approach was employed to refine the calibration parameters (Table 5).

**Table 5:** Calibration coefficients and correlation matrix.

	Value	Error	F	Cx	Cy	K1	K2	P1	P2
F	**5821.76**	0.12	1.00	− 0.05	− 0.59	− 0.11	0.18	0.00	− 0.31
Cx	**−1.28316**	0.14		1.00	0.08	− 0.03	− 0.00	0.95	0.11
Cy	**22.4569**	0.12			1.00	− 0.11	− 0.00	0.08	0.81
K1	**0.0025053**	5.9e-05				1.00	− 0.90	− 0.05	− 0.14
K2	**0.0072501**	0.00015					1.00	− 0.00	0.01
P1	**− 0.000518583**	7.8e-06						1.00	0.15
P2	**0.000425885**	6.5e-06							1.00

**Fig. 13:**
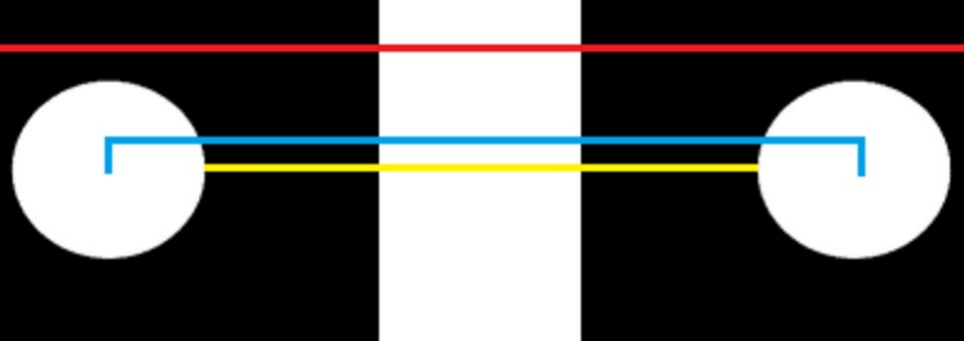
Measuring distances: left to right (red), right to left (yellow), and center to center (blue).

Next, as mentioned, to scale the model and transfer it to metric space, distances between control points were measured using an industrial digital caliper. This involved three-step readings: left to right, right to left, and center to center of the targets (Fig. 13), with an accuracy of 5 to 10 microns.

**Fig. 14:**
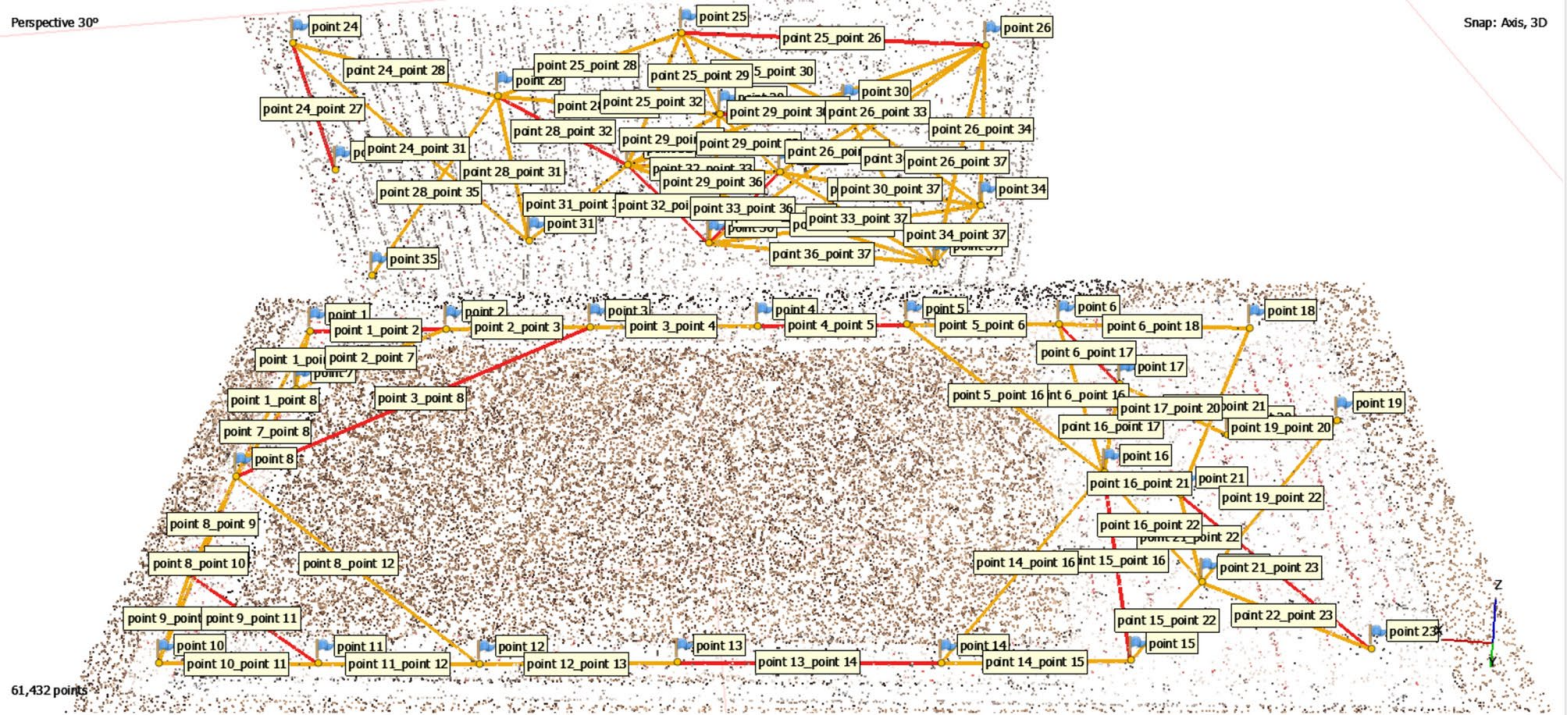
Design of control lengths (in red) and check lengths (in yellow) on the working section body.

Fifteen control lengths and 55 check lengths were designed on the body for evaluating the accuracy (Fig. 14). The distribution of scale bars was meticulously planned to ensure comprehensive coverage of the entire working area and it leads to choosing suitable control lengths for scaling the model and examining errors with check lengths.

Subsequently, the Semi-Global matching (SGM) algorithm^40^ was used for 3D reconstruction of the soil point clouds separately for each epoch’s images. The Semi-Global Matching (SGM) algorithm is a technique employed for stereo vision and depth map estimation. It integrates local and global matching methods to enhance the accuracy of disparity maps, which depict the positional differences between corresponding pixels in two stereo images.

**Fig. 15.**
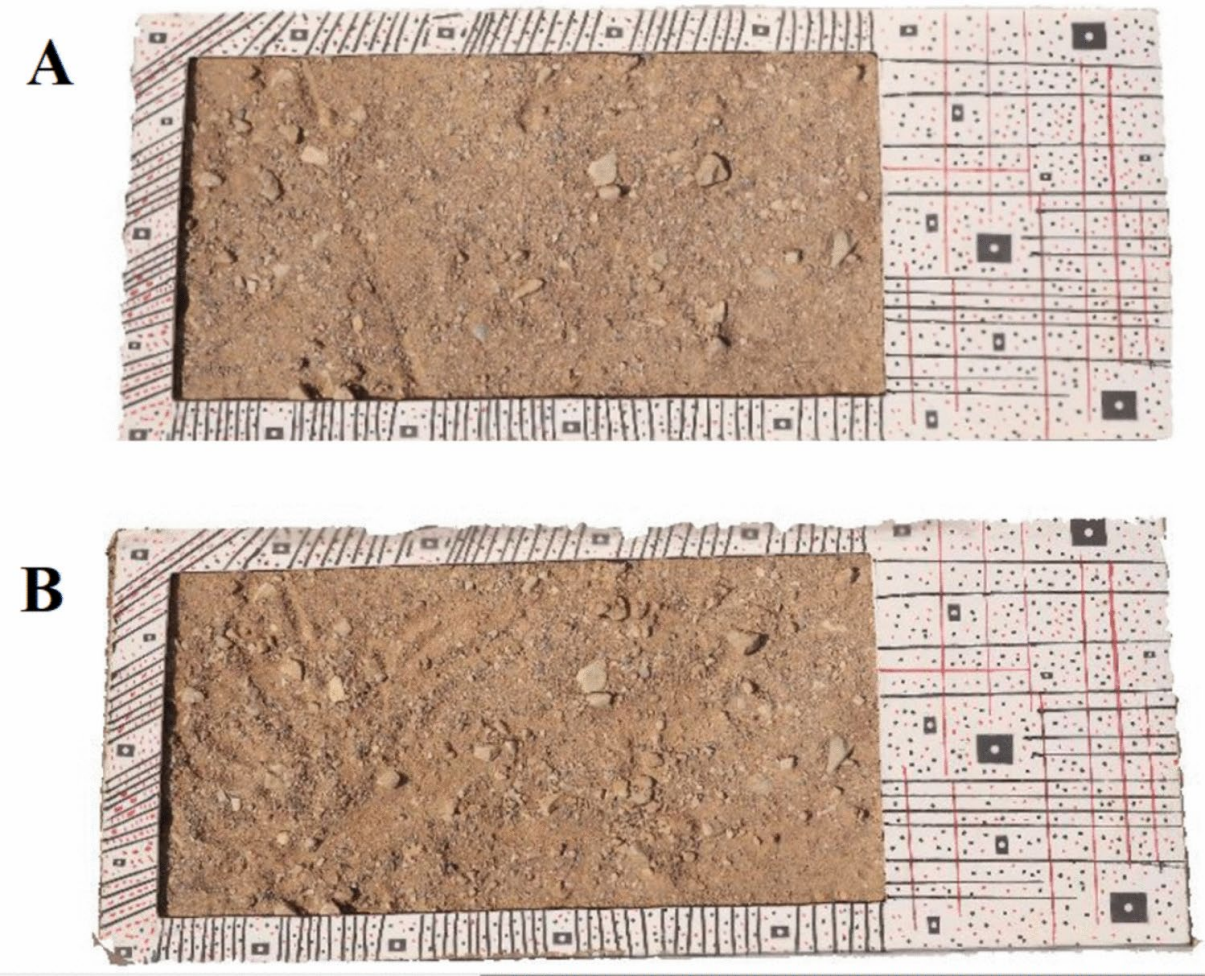
3D models generated from both epochs. A: first epoch and B: second epoch which show the soil sample before and after wind erosion.

By performing the bundle adjustment calculations in this way, after the 3D reconstruction of the point clouds of both epochs, there is no need to perform the registration process of the point clouds, and after that, the final 3D model for both epochs was created using Metashape software version 1.8.5 (Fig. 15).

As mentioned, with the same processing of both data series at once, the bundle adjustment is calculated for both modes at once and only for 3D reconstructions such as 3D point clouds and 3D models only considering the images related to each epoch. 3D color point clouds are created for each epoch, the calculation that was performed in the previous step was corrected, and the images related to the epoch section were considered. Therefore, both point clouds are completely matched.

According to the accuracy of the check lengths, an accuracy of 0.175 mm was calculated in the form of length calculation error. Additionally, Some check lengths which calculation difference between calculated and measured values are shownin Table 6.

**Table 6:** Some check lengths estimations and the corresponding errors from comparisons with measured values.

Label	Distance (mm)	Error (mm)
point 1-point 7	75.1448	0.222344
point 1-point 8	172.039	0.303559
point 7-point 8	99.1077	0.270196
point 8-point 9	94.8059	0.373384
point 8-point 10	169.718	0.215732
point 9-point 10	75.132	0.129525
point 2-point 7	134.934	0.279373
point10-point 11	113.665	0.169989
point 8-point 12	284.329	− 0.0162732
point 25-point 29	76.1636	− 0.283922
point 29-point 33	71.8	− 0.129954
point 29-point 36	130.599	0.0692409
point 36-point 37	193.797	0.124851
point 34-point 36	230.407	− 0.0976335
point 26-point 36	290.297	0.0296511
point 33-point 34	167.718	0.0727206
point 33-point 37	161.212	0.164356
point 26-point 33	195.163	0.110476
point 30-point 34	147.069	0.021104
point 30-point 37	174.743	− 0.0373003
point 34-point 37	70.4946	− 0.225439
point 26-point 34	150.472	− 0.0230445
point 26-point 37	213.524	− 0.20584
**Total**		**0.174855**

**Fig. 16:**
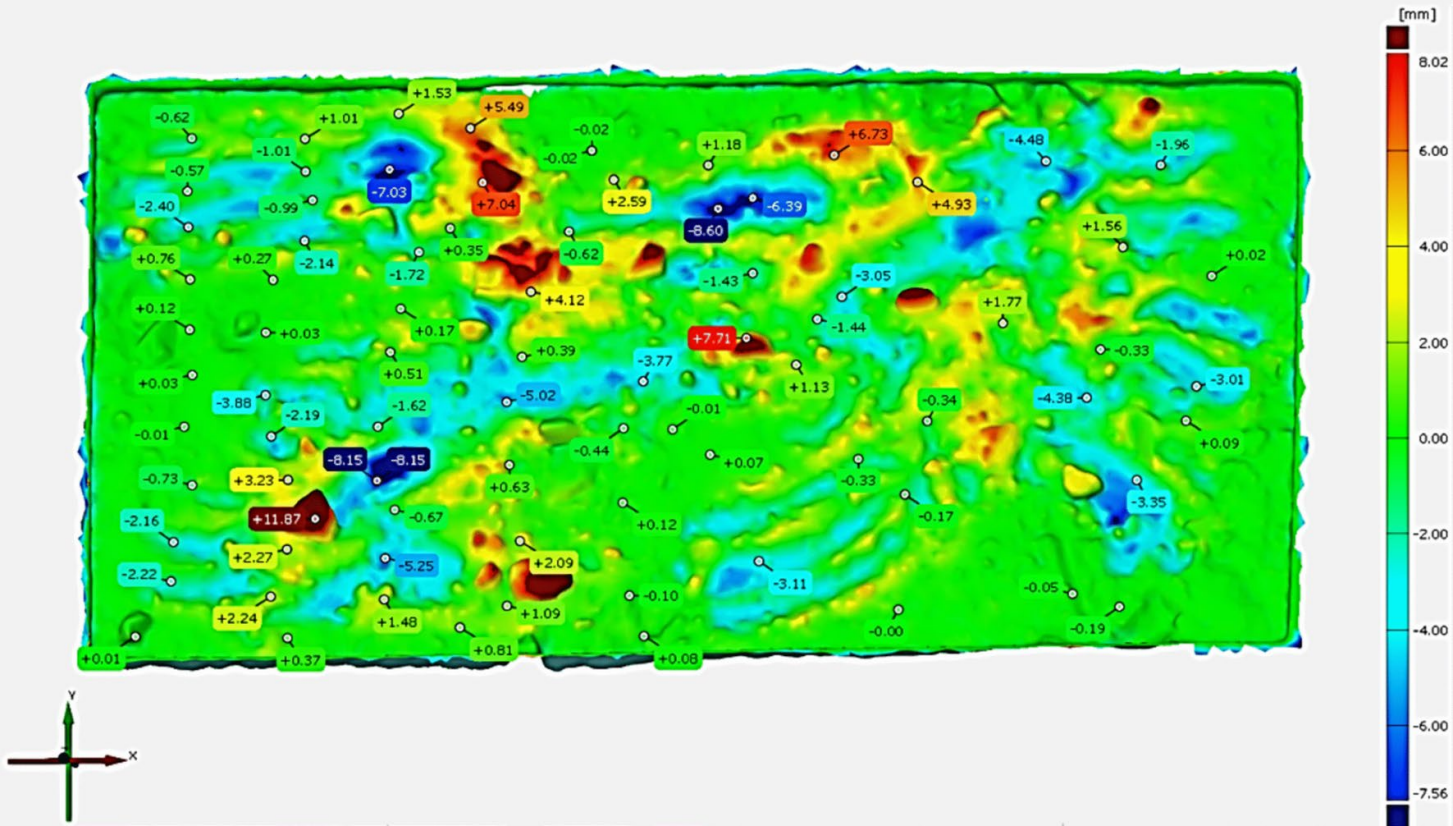
Visualization of the differences between 3D models as point clouds for both epochs in GOM software (version, 2018.0.146193). The numbers in this figure show the microtopogphy changes (in mm) during two epochs wich negative and positive numbers represent the erosion and sedimentation points, respectively.

Based on the performed tests, the extent of changes in the GOM software (version, 2018.0.146193) was calculated, and the pointwise analysis outputs of various soil parts are shown in Fig. 16. GOM Inspect is a software for analyzing 3D measuring data from fringe projection or laser scanners, coordinate measurement machines (CMM) and other measuring systems^41^. The software GOM was used to evaluate the amount of changes that occurred at each point, which could be analyzed based on soil type and environmental parameters. In Fig. 16, the numbers indicate the extent and direction of soil change, where a positive value ( +) signifies an addition of soil to the area, and a negative value ( −) indicates soil removal.

Due to limited access to precise equipment such as 3D laser scanners or other high-accuracy scanning devices, which are required to achieve better accuracy for robust validation compared to photogrammetry, an alternative validation method was necessary for our tests. Therefore, we decided to use an accurate caliper, as previously mentioned, to validate control and check lengths. The accuracy of this method was evaluated based on the error in estimating the check lengths.

It is important to note that various factors influence the final accuracy of the results, including environmental conditions, lighting, camera position, orientation, and direction. Furthermore, ensuring the stability of the soil between two epochs (with no soil movement observed during this test) was essential. These tests were conducted under conditions free from significant wind or adverse weather, as such factors could considerably affect accuracy.

**Fig. 17:**
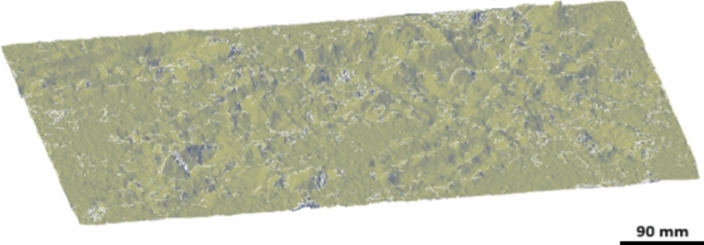
Visualization of contour lines and 3D mesh of point clouds for epochs in Autodesk Autocad Civil3D 2015 software.

Next, volumetric operations of the soil were calculated. Table 7 shows the number of cut and fill operations based on the point clouds created for each epoch. These point clouds were used to create 3D surfaces and generate elevation contour lines (microtopography). Then, by comparing the 3D surfaces created, the amount of soil change (earthwork volume) was calculated in each dimension using Autodesk Autocad Civil3D 2015 software^42^ which offers a better and easier way to design, analyse and document civil engineering projects (Fig. 17). According to Table 7, the difference in soil volume between the second epoch and the first epoch, calculated by Civil3D software, is 7756.75 mm^3^.

**Table 7:** Volume of cut-and-fill operations (cut/fill report).

Name	Area (mm^2^)	Cut (mm^3^)	Fill (mm^3^)	Net (mm^3^)
Soil sample	168,806.25	143,694.23	135,937.48	7756.75 < Cut >

It is important to note that the reported results are not definitive, as the conditions for data acquisition and the designed network may change. If the network design, image acquisition, camera calibration, and image processing are conducted under optimal conditions, results comparable to those of this project can be obtained.

### Removal of anemometer

**Fig. 18:**
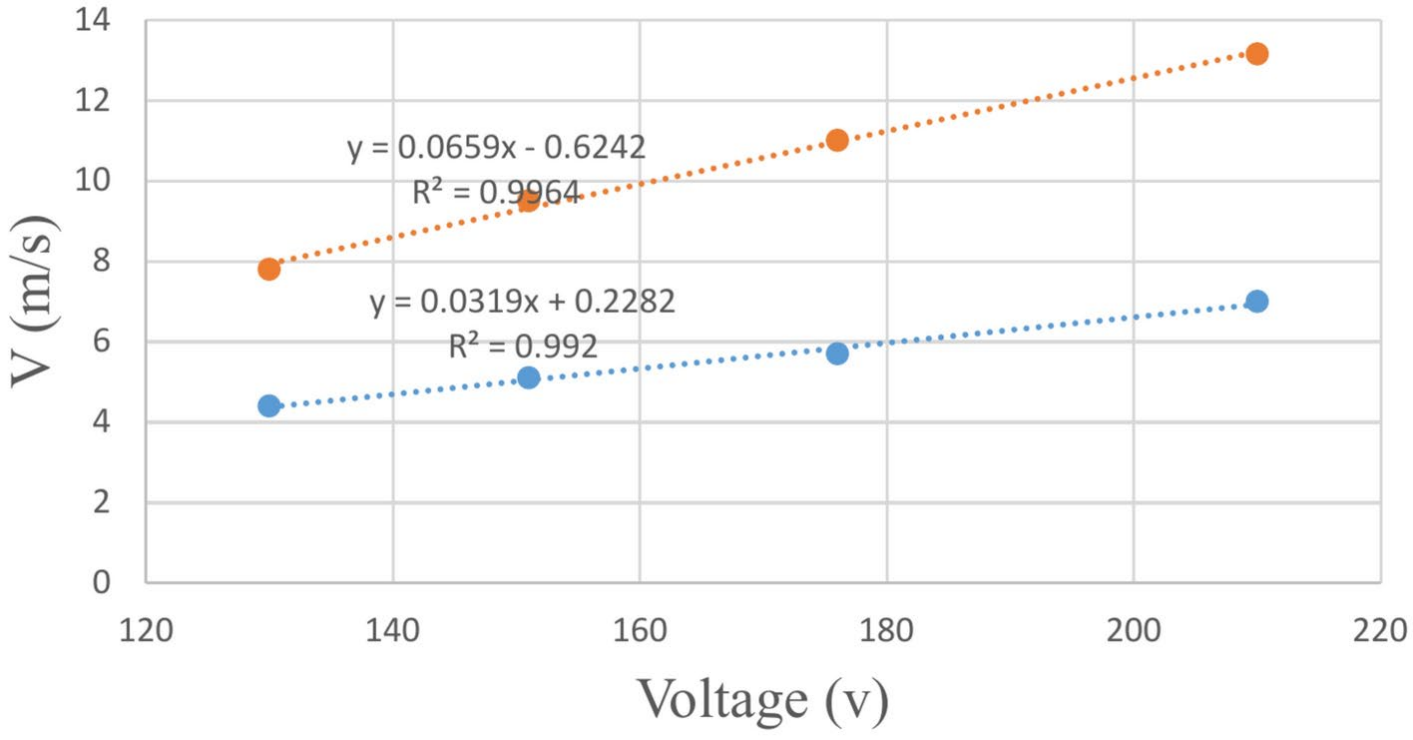
Relationship between the wind speed and the input voltage to the fan (blue and brown lines show the velocities at 5 and 40 cm above the soil sample surface, respectively).

The fan motor speed is altered to generate winds at desired velocities. Typically, in most wind tunnel devices, the wind speed is measured using an anemometer^3,5,9^ or pitit tubes^20^. However, in this project, the wind speed is calculated by establishing a relationship between the fan speed and the input voltage to eliminate this process for user friendliness. To achieve this, it is essential to estimate the relationship between the output voltage of the inverter and the produced wind speed. Therefore, initially, wind speeds in the working section were measured using an anemometer at various voltage levels. The results of these measurements demonstrated a highly linear relationship between the output voltage of the inverter and the wind speed in the working section (Fig. 18).

As mentioned, some research^3,5,9,20^ has utilized an anemometer to measure wind speed in the working section as a traditional method. In most of wind tunnles, an anemometer was installed in the working section and the measured speed values were read in different ways, or these values were given to a control system so that appropriate adjustments were made in the fan output. In the proposed scheme of this paper, the relationship between the output voltage of the inverter and the wind speed in the working section was calculated using the wind speed values measured at different heights and conditions (in Fig. 18, the equations are presented for two different heights of 5 and 40 cm). Therefore, by using these equations, all the wind speeds produced by the fan are covered, and it is only enough to put the output voltage of the inverter in the equations, where each voltage corresponds to a specific wind speed. Unlike other methods, this method does not require the permanent installation of an anemometer in the working section and enables easy calculation of the wind speed based on the inverter’s output voltage using a regression relationship. This innovation eliminates the need for an anemometer during device operation, which is one of the acheivments of this project.

As mentioned before, in order to obtain the wind speed according to the inverter voltage, a calibration process has been performed for each height and some equations have been obtained. These equations are specific to the corresponding heights. The equations for two heights of 5 and 40 cm above the soil sample surface are given in Fig. 18. This is despite the fact that the equations for any height can be obtained by performing similar experiments and obtaining the relevant equations.

Each of the mentioned equations to obtain the wind speed is only valid for the condition that all the electrical equipment and tunnel channel are in healthy condition. So, if any of the equipment is not working properly, the resulting equations may not correctly calculate the actual output. Considering that the equipment aging and some faults can reduce the efficiency of the equipment or lead to their failure, two periods of recalibration are recommended according to two indicators (time period and number of times the wind tunnel is used for soil erosion analysis). A one-year time index is considered for the first indicator and 30 times is considered for the number of times the tunnel is used. Therefore, if each of these indicators exceeds its threshold, recalibration can be performed in order to maintain accuracy.

## Discussion and conclusion

The main objective of this study is to describe and explain the design and capabilities of the device invented by the authors in the field of wind erosion assessment and soil loss. Therefore, the achievements of this device are analyzed in various sections. The achievements of the photogrammetry section include:

- Creation of a scaled metric coordinate system
- Elimination of the registration process
- High convergence accuracy in network strength
- Manual target design for creating coordinate systems
- Production of 3D models and control points, and checks for applying the scale and evaluating the condition.

Considering the photogrammetry outputs and performed adjustment calculations, photogrammetry can be considered a cost-effective and accurate method for creating 3D models and control points. However, limitations in controlling the accuracy of 3D reconstruction were significant challenges in this project because there was no access to a more accurate system for reference data extraction. For this reason, the project relied on scale bars and the accuracy of adjustment calculations. It is worth mentioning that for the production of control and check lengths, a robust network design was used to reconstruct control points of scale bar targets on the body. The adjustment process of both epochs simultaneously and the computational error of the triangulation process estimated on the check points were 0.175 mm, demonstrating the high network strength of the photogrammetry. Furthermore, the separation of epochs for 3D reconstruction allowed for the complete alignment and registration of 3D models and control points, eliminating the need for further registration. So, due to removing registration process for two epochs with proposed method, it causes improved the accuracy of change calculations in different areas of the soil based on soil type and environmental parameters.

According to Table 7, an area of approximately 94% (168,806 out of 180,000 square millimeters, which is equivalent to the working section area) is simulated in 3D, and the remaining area (approximately 6%) is related to the sides of the working section.

While in most previous studies on calculating eroded soil masses, sediment traps have been used^5,8,9,14,17^, in some more recent studies, soil volume was measured using laser scanning^43,44^. However, according to previous investigations^30,31,35,36,39^, photogrammetry has not been used for soil volume calculations in wind tunnels. The use of this technique not only eliminates the need for sediment traps but is also more cost-effective than the use of laser scanners. The price of the equipment used for the photogrammetry section of the device is approximately one-sixth the price of the laser scanner used in a previous study^39^, which is referred to as an affordable device (€3000).

Another feature of this device is its portability, which results from its small size, ability to separate parts, low weight, and strength of its various components. Although the lengths of other similar devices^8,12,18–21,26^ range from 5 to 15 m, primarily due to the length of the sediment trap, this device does not require the use of a sediment trap or the collection of eroded soil because of the calculation of soil volume using the difference in soil volume in images before and after the test. Another advantage is the complete transparency of the working section, achieved through the use of transparent Plexiglas sheets in the body. This feature allows easy determination of the threshold wind erosion velocity (the lowest velocity for soil particle movement), which is a critical parameter in wind erosion studies^45^.

Another advantage of this device is the minimal use of connections, achieved by bending the Plexiglas sheet (rather than cutting it), which increases the device’s lifespan. Another important achievement is the removal of the anemometer, which has been achieved by estimating the linear relationship between the output voltage of the inverter and the wind speed at the entrance of the working section. For this research study, in the condition that all components, including the inverter, work correctly and without failure, there is no limit to using the equations related to the relationship between the wind speed in the working section and the output voltage of the inverter to provide different wind speeds. However, in some situations, including the lack of complete health of any electrical equipment such as the fan and the inverter and other equipment such as the wind tunnel channel, these equations cannot be used. Also, when there is a need to change the wind speed range to check soil erosion, a fan with new specifications should be replaced, in which case it will be necessary to obtain new equations according to the new conditions. According to the mentioned cases, this device can be applied in cases such as quantitative measurements of soil erodibility, in mulch and soil cover test laboratories, in wind erosion dynamics laboratories, for preparing maps of land sensitivity to erosion, and for sedimentation measurements.

## Data Availability

The data used to support the findings of this study are available from the corresponding author upon reasonable request.
